# Protein array identification of protein markers for serodiagnosis of *Mycobacterium tuberculosis* infection

**DOI:** 10.1038/srep15349

**Published:** 2015-10-20

**Authors:** Fangbin Zhou, Xindong Xu, Sijia Wu, Xiaobing Cui, Lin Fan, Weiqing Pan

**Affiliations:** 1 Institute for Infectious Diseases and Vaccine Development, Tongji University School of Medicine, Shanghai, China; 2 Clinic and Research Center of Tuberculosis, Shanghai Key Lab of Tuberculosis, Shanghai Pulmonary Hospital, Tongji University School of Medicine, Shanghai, China; 3 Department of Tropical Infectious Diseases, Second Military Medical University, Shanghai, China

## Abstract

The lack of effective and accurate diagnostic tools contributes to the high prevalence of tuberculosis (TB) worldwide. The current serodiagnostics for TB are inadequate mainly due to lack of TB-specific antigens with highly accurate diagnosis. In the current study, we aimed to identify novel diagnostic antigens using glutathione S-transferase (GST)-fusion protein technique. We determined the reactivity of these recombinant proteins arrayed in solution and on GSH-immobilized microplates with TB patient sera. Of 409 TB proteins produced, ninety-two yielded seropositive reactions, fourteen including eight novel proteins showed strong immunoreactivity. Further, six were selected and constructed as a multiple-antigen combination set through analysis of various combinations. A comparative study of the multiple-antigen combination set and a commercially available kit revealed that the combination set showed 66.3% (95% CI 60.5–71.8) sensitivity, which was significantly higher than that of the commercial kit [31.6% (95% CI 26.3–37.3)]. The specificity of both methods was similar at 89.6% (95% CI 83.3–95.4) and 90.6% (95% CI 83.0–95.6), respectively. This study provides a set of novel diagnostic protein markers with great potential for the development of novel diagnostic tools for active TB.

Tuberculosis (TB) is a chronic infectious disease caused by infection with *Mycobacterium tuberculosis.* It remains a serious global health problem with 8.6 million new cases and 1.3 million deaths reported in 2012^1^. Moreover, nearly one-third of the world’s population is latently infected with *M. tuberculosis*, of which approximately 10% develop active TB in their lifetime^2^. In addition, the emergence and spread of the multidrug-resistant TB is threatening the global control program^3^,^4^,^5^.

The lack of effective diagnostic methods is a major contributing factor in the high disease prevalence. The sputum smear and culture tests are the traditional gold standard methods for TB diagnosis; however, the former exhibits low sensitivity particularly in children and immunocompromised individuals, while the latter requires a very long cultivation period (6–8 weeks)^6^. The tuberculin skin test (TST) is the only screening test for latent *M. tuberculosis* infection, but it exhibits extremely low specificity and high cross-reactivity with other species of mycobacteria^7^.

TB diagnosis has been improved by the recent development of methods such as nucleic acid amplification (NAA) tests, rapid culture methods, T-cell-based interferon-γ release and advanced imaging techniques^8^. However, the use of these methods in developing countries where majority of TB cases occur is limited by cost. In addition, these techniques are not suitable for large-scale surveillance and screening tests. Therefore, more accurate and economic diagnostic tools for TB are urgently required.

Rapid, point-of-care diagnostics that allow highly accurate diagnosis of active TB in the field are urgently required. Immunodiagnostic detection of TB antigens or their antibodies, which is simple, cheap, robust and easily implemented, represents an attractive option for this purpose although the sensitivities of the currently available options are quite variable ranging from 0.09% to 59.7%^9^. Recent efforts involving high-throughput screening of the entire *M. tuberculosis* proteome have identified some promising diagnostic antigens, including the 38-kDa antigen, which has been used in some commercial immunodiagnostic kits^10^,^11^. Moreover, the combination of several antigens significantly improved the diagnostic sensitivity^12^,^13^.

The availability of *M. tuberculosis* genome sequence information along with the corresponding proteomic datasets has facilitated the comprehensive, systematic and unbiased identification of novel antigenic proteins at a whole proteome scale^14^,^15^. Although a large number of antigens have been identified for the detection of circulating antibodies to *M. tuberculosis* and evaluated for their diagnostic value alone or in combination, their use is limited by low sensitivity and specificity^16^,^17^. Thus, further studies are needed to identify more effective diagnostic protein markers. Many previous screenings of diagnostic antigens either used denatured recombinant protein expressed by *Escherichia coli*^18^, thus, increasing the possibility that some conformational epitopes may be lost, or used native materials extracted from the bacteria or carrier proteins^19^,^20^,^21^, although folding, probably limited by the low level expression of the immunologically meaningful proteins, such as secreted proteins and transmembrane proteins^22^,^23^, therefore undetected. It was noteworthy that immunodominant antigens identified in vast literatures were enriched for secreted and transmembrane proteins^24^,^25^,^26^,^27^. Two recent proteome-wide approaches for identification of potential *M. tuberculosis* protein biomarkers revealed that the subset of the proteome targeted by a human immune response was predominated by secreted and transmembrane proteins^18^,^28^. These conclusions were consistent with much earlier diagnostic works which had identified some well-known immunodominant antigens of *M. tuberculosis*, including ESAT-6, the 38 kDa antigen, CFP-21 and MPT64 that were secreted by the bacteria during infection^29^,^30^ and LprA and HspX that were extracted from isolated plasma membrane^31^. Moreover, though it was widely recognized that protective immunity against intracellular bacteria such as *M. tuberculosis* was primarily elicited by T-cell-mediated response, antibodies were still produced during infection and secreted and transmembrane proteins had been identified to be targeted by B-cells in other intracellular bacterial species, including *Chlamydia*, *Ehrlichia* and *Listeria*^32^,^33^,^34^. As a consequence, we focused our search for serodiagnostic antigens on secreted and transmembrane proteins of *M. tuberculosis*. Recently, we developed a glutathione *S*-transferase (GST) fusion protein assay, in which each protein is refolded, arrayed in solution and at similar amounts. This technique could potentially identify antigens present at low levels, or antigens with short half-lives, and has been successfully applied to identify diagnostic markers for other pathogen^35^. In the current study, we used this technology to generate over four hundred *M. tuberculosis* secreted and transmembrane proteins, which we analyzed using a large number of serum samples from TB patients. We discovered several novel diagnostic antigens and constructed a multiple-antigens combination set that significantly increased the sensitivity and specificity of immunodiagnostic testing of TB infection in humans.

## Results

### Production of recombinant GST-TB fusion proteins

A total of 239 putative secreted proteins and 358 transmembrane proteins were predicted from 3,924 *M. tuberculosis* open reading frames (ORFs) using bioinformatics tools. We excluded 118 transmembrane proteins, of which the protein sequence of the largest extracellular domain was less than 50 residues. In addition, we selected 91 latent-associated proteins and 129 RD (region of difference) proteins, which were absent from BCG but present in *M. tuberculosis*.

**Figure 1:**
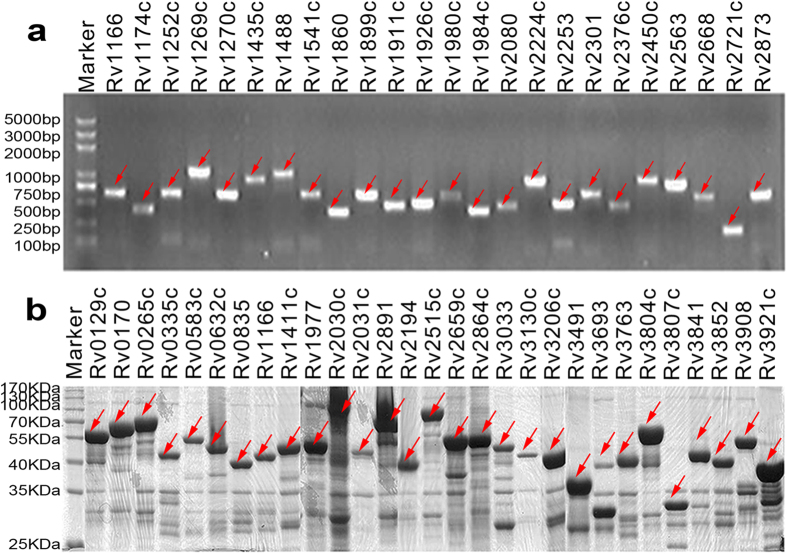
PCR amplification and expression of *M. tuberculosis* ORFs. (**a**) A total of 699 unique pairs of primers based on bioinformatics analysis were designed for PCR amplification and 630 (90%) ORFs of the correct size were successfully obtained. The PCR products were separated by agarose gel electrophoresis. The corresponding ORF proteins are annotated at the top of the figure according to the *M. tuberculosis* H37Rv nomenclature (http://genolist.pasteur.fr/TubercuList/). (**b**) All samples were separated by 12% SDS-PAGE and Coomassie blue stained. The corresponding protein identities are annotated at the top of the figure. Bands corresponding to the correct size of ORF and proteins are indicated by red dots. The gels were cropped and run under the same experimental conditions.

The full-length sequences of secreted proteins, RD proteins, latent-associated proteins and the largest extracellular domain of transmembrane proteins were amplified by PCR (Fig. 1a). A total of 441 genes were successfully cloned from the resulting PCR products into the pGEX-His vector (Supplementary Table S1). Western blot analysis showed that a total of 432 (97.9%) GST-TB fusion proteins were successfully expressed in *E. coli* (Fig. 1b and Supplementary Table S5), and of these, 389 (85.3%) were mainly expressed in inclusion bodies.

**Figure 2:**
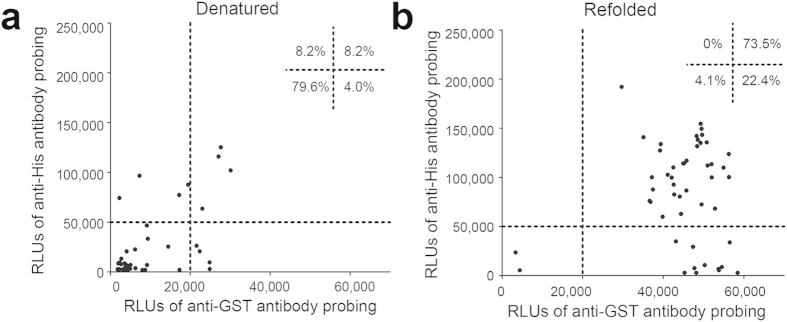
Validation of the proteome screening platform reliability. Distribution of signal intensities of refolded (**a**) and denatured (**b**) GST-TB fusion proteins were obtained by anti-GST and anti-polyhistidine-tag antibody probing. RLUs = relative light units.

Correct refolding of the GST-TBs fusion proteins expressed in inclusion bodies is essential for their binding to the GSH-immobilized microplates. When detected by ELISA using anti-GST-tag and anti-polyhistidine-tag mouse monoclonal antibodies, the relative light units (RLUs) of most of the refolded proteins were significantly higher (Fig. 2b) than those of the denatured proteins (Fig. 2a), indicating that the recombinant proteins were successfully refolded and most were full-length. Moreover, chemiluminescent ELISA indicated that 409 (94.7%) of the 432 GST-TB proteins were successfully bound to the GSH-immobilized microplates.

### Screening of 409 GST-TBs with TB patient serum samples

**Figure 3:**
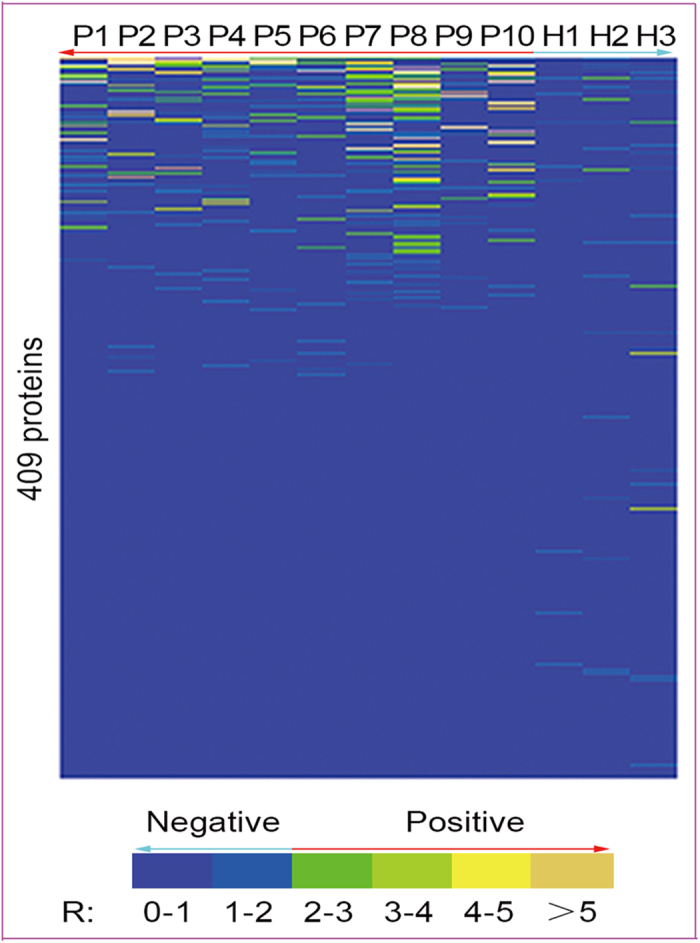
Screening of the GST-TBs with tuberculosis patient sera. Ten TB patient sera and three healthy control sera were used for initial screening. The reactions of the serum samples to 409 putative *M. tuberculosis* proteins were determined by chemiluminescent ELISA. The RLUs were detected by the chemiluminescent reader SpectraMax M5. The ratio of RLUs of a human serum to a GST-TBs and the same serum to GST control was calculated using the formula: R = (RLUs of GST-TBs − RLUs of PBS)/(RLUs of GST − RLUs of PBS). GST-TBs with R ≥ 2 were considered to indicate seropositivity.

Initial screening of the interactions of the 409 GST-TB fusion proteins with 10 TB patient serum samples was assessed by chemiluminescent ELISA; three healthy serum samples were used as negative controls. Of 409 GST-TBs, 92 had seropositive reactions (Fig. 3, Supplementary Table S6). The number of reactive antigens varied with different serum samples. Of the 92 antigens, 24 (26.1%) and 43 (46.7%) interacted with the serum samples from Patients 7 and 8 respectively, while only 12 (13.0%) and 6 (6.2%) interacted with serum samples from Patients 5 and 9.

Of the 92 seropositive antigens, 55 reacted with only one TB patient serum sample, while 14 reacted strongly with at least two samples and showed no cross-reactivity with serum samples from healthy controls. Of these, eight were novel protein markers for TB serodiagnosis. The 14 proteins were selected as candidate protein markers for further analysis and the proteins without the GST-tag were re-produced in *E. coli* as shown in Supplementary Figure S1 and Supplementary Table S2.

### Assessment of 14 candidate diagnostic antigens

**Figure 4:**
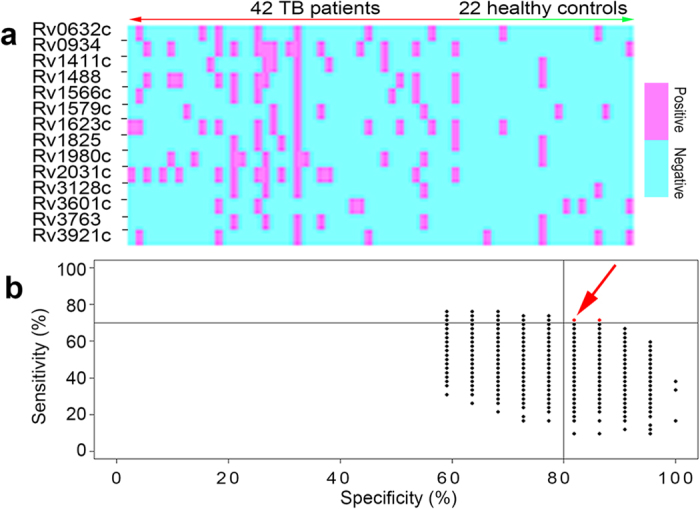
Reactivity of the 14 selected TB proteins. (**a**) Their responses to the panel of 42 archived infected serum samples and 22 healthy controls are displayed in a heat map. (**b**) The caret package in R statistical software was used for the analysis. The sensitivities and specificities of variable combinations of proteins (range, 2–14) were calculated. Each dot represents one combination (combinations with identical sensitivities and specificities are overlapping). Combinations with higher sensitivities and specificities (more than 0.7 and 0.8, respectively) are marked with red dots.

The potential of the 14 most reactive antigens as protein markers for the serodiagnosis of active TB was evaluated using a panel of 64 randomly archived serum samples (42 infected samples and 22 uninfected samples). The specificities of all the recombinant proteins exceeded 80% (81.8–100%), whereas their sensitivities ranged from 9.5% to 33.33% (Fig. 4a and Supplementary Table S3). Clearly, no single antigen was representative of the antibody profiles of all TB patients; therefore, a combination of various antigens is required to improve the diagnostic potential.

The sensitivities and specificities of variable combinations of proteins (2–14) were analyzed statistically. A total of 16,369 different combinations were shown (Fig. 4b). A total of 207 different combinations of sensitivity and specificity were identified, of which, a combination of six proteins (the 38kDa antigen, LprG, Rv1566c, Rv1623c, MPT64 and HspX) provided the best performance (71.4% sensitivity with 86.4% specificity) and was selected for comprehensive evaluation (Supplementary Table S3). Among the six proteins, Rv1566c and Rv1623c were novel proteins for TB serodiagnosis.

### Diagnostic validation of the multiple-antigen combination set

The sensitivity and specificity of the multiple-antigen combination were further analyzed for TB serodiagnosis using serum samples from 96 patients infected with TB and 49 healthy individuals (Supplementary Figure S2 and Table 1). All antigens had a specificity of 95.9% or greater, while the sensitivities of the six individual antigens varied from 15.6% to 33.3%. Of the infected serum samples, 67.7% (65/96) recognized at least one antigen, while only 6.2% (4/65) recognized all the antigens and 55.4% (36/65) reacted only with the discrete antigens. Of these, seven serum samples interacted with the 38 kDa antigen alone, two interacted with LprG, three with Rv1566c, eleven with Rv1623c, seven with MPT64, and six with HspX. The combination of the six antigens significantly increased the sensitivity to 67.7% with a specificity of 91.8% (Table 1). Thus, the combined antigens were identified for further validation as serodiagnostic markers of TB.

### Comparison of the combination test with a commercially available kit

To examine the potential diagnostic value of the multiple-antigen test, we collected an additional 384 serum samples, including 288 infected serum samples from the patients with clinically diagnosed TB during the period from September 2012 to October 2014. We compared the specificity and sensitivity of the combined antigens with a commercial diagnostic test (TAID-Kit, Tuberculosis Antibody (IgG) Detection Kit (ELISA), Shanghai). Of the 288 TB patients, the combined antigen test identified 191 as seropositive (66.3% sensitivity), whereas the TAID-Kit diagnosed only 91 as seropositive (31.6% sensitivity). The specificity of both methods was similar (89.6% and 90.6% respectively). Moreover, the combined antigen test provided higher accuracy compared with the TAID-Kit (72.1% vs. 46.4%) (Table 2).

## Discussion

The WHO has developed a new six point Stop TB Strategy (the Stop TB Partnership's Global Plan to Stop TB 2006–2015), which aims to achieve the gradual elimination of TB and considerable effort has been directed towards preventing its high prevalence^36^. However, none of the current strategies are likely to achieve this level of disease control; consequently, the identification of new diagnostic biomarkers presents a priority in TB research. In this study, we used the recently developed high-throughput GST-fusion protein array technology to generate over 400 TB proteins. Fourteen highly reactive proteins were identified by screening with TB patient serum samples, eight of which were novel. These results provide experimental evidence of the immunogenicity of novel TB proteins that are suitable for the development of valuable serodiagnostic tools.

The availability of MTB genome sequences provides the opportunity for the systematic identification of diagnostic protein markers and vaccine candidates at a genome-wide scale. However, limited progress has been made, mainly due to difficulties in limiting the bias introduced by differences in the amounts of proteins present in such screening procedures. Many previous studies based on ‘omics’ approaches perform suboptimally and have respective drawbacks. For example, 2-D gel electrophoresis and liquid chromatography followed by mass spectrometry (MS) used native samples extracted from *M. tuberculosis* culture supernatant^20^,^22^,^24^. However, because of the complexity of protein extract preparation and separation, the success of these investigations were limited by the abundance of the expressed proteins, with immunologically relevant proteins, such as secreted proteins and transmembrane proteins, often present in low abundance, and therefore undetected. On the other hand, one recent proteome-wide approach was developed to identify serodiagnostic antigens of *M. tuberculosis* at an unbiased way^18^. However, the expressed recombinant proteins dissolved in urea were at denatured state, thus increased the possibility that important conformational epitopes were lost. Other recently developed technologies involve a protein microarray method named Nucleic Acid Programmable Array (NAPPA), in which functional proteins are transcribed and translated *in situ* directly from printed full-length cDNAs just-in-time for assay^37^. The recently modified NAPPA method further shows multiple advantages over others such as highly consistent display levels, flexibility, high throughput, and so on. Moreover, this method uses purified cDNA as the key substrate, which is much easier to prepare, print, quantify and store than proteins^38^. It exhibits the great potential and capacity to screen of candidate targets and has successfully applied to other human diseases^39^,^40^. In this study, we use an indirect ELISA-based GST-fusion protein array technique, which integrates multiple advantages as follows: (1) High throughput screening: this technology allows us to produce a large number of GST-TB fusion proteins that are bound to the 96 or 384 GSH-immobilized plates for screening, which provides a possibility of the systematic identification of novel protein markers for serodiagnostics at an ORFeome scale; (2) Faster and less labor-intensive: no purification procedure is needed for the GST fusion proteins in the bacterium extracts that are directly added to the GSH-immobilized plates for the binding through the interaction of GST with its substrate glutathione; (3) Potential identification of the protein markers at low expression at nature condition: the immunologically meaningful proteins such as secreted and transmembrane proteins normally presenting at a low level expression at nature condition could be produced at a large volume and high-quality of the products using this standard technology and potentially identified as diagnostic protein markers; (4) Correct refolding of the GST fusion proteins: binding of the GST-TB proteins to the GSH-immobilised plates means that all the bound fusion proteins could refold correctly. In addition, the recombinant proteins expressed in *E. coli* particularly those in inclusion bodies are essential for refolding before their binding to the GSH-immobilized microplates. We have identified a universal refolding buffer C7 from the iFOLD Protein Refolding System 1 for the refolding of the GST inclusion bodies^35^. We demonstrated the correct refolding of the GST-TB proteins by comparison of the interactive signal intensity with both anti-GST and anti-polyhistidine-tag antibodies with that of the denatured proteins (Fig. 2a). All of the refolded proteins but twenty-three bound successfully to the plate and were detected by the anti-GST monoclonal antibody (Fig. 2b), indicating correct folding. The remaining twenty-three proteins failed to bind to the plate probably due to incorrect folding. The ability to generate correctly refolded recombinant proteins is critical for the discovery of ideal diagnostic proteins. Using this system, we successfully identified fourteen highly immunoreactive proteins, eight of which were novel and had not been reported previously as diagnostic candidates. The detection of previously established antigens in addition to the relatively high rate of novel antigen detection confirms the efficacy of this approach. The ELISA-based GST-fusion protein array technique has also been successfully used in the identification of antigens for the serodiagnosis of *Schistosoma japonicum* infection^35^. Thus, the results of our study indicate the potential application of this approach for serodiagnosis of other bacterial and parasitic diseases.

A total of 409 GST-TB fusion proteins were produced and bound to the GSH-immobilized plates. Serum samples from active TB patients and healthy controls were used to screen of the putative GST-TB fusion proteins. The *M. tuberculosis* ORFeome reactivity of sera from different individuals was distributed among three distinct groups. The first, and smallest group, which comprises roughly 3.4% (14/409) of the ORFeome, includes antigens that are most frequently recognized only during active TB. Of these antigens, Rv1488, Rv1566c, Rv1579c, Rv1623c, Rv1825, Rv3128, Rv3601c, and Rv3921c were newly identified. A second, larger group includes additional, frequently recognized antigens as well as proteins that are rarely detected. Together, the first two groups of proteins define the immune-ORFeome of *M. tuberculosis*, which is approximately a quarter of the size of the total ORFeome established in this study. These targets are rich in secreted and transmembrane proteins. The remaining, and largest, group of the ORFeome contains proteins that do not react with TB sera.

Previous evaluations of serological responses indicate a heterogeneous antibody response to TB antigens^13^,^41^,^42^, which is consistent with our results. While many TB patient sera reacted with different antigens, some sera were exceedingly restricted in their reactivity (Fig. 4a). Some sera reacted almost exclusively with discrete antigens, while other sera reacted with almost all antigens. However, most sera reacted with a variety of multiple antigens. Variations in specific antibody responses to TB antigens in different individuals may be linked to human leukocyte antigen (HLA) phenotype^43^,^44^, bacillary load and disease progression of the patient populations^28^. No single TB antigen has so far been identified as a useful serodiagnostic marker due to the complexity of the human immune response to TB antigens. To overcome this issue, a combination of several recombinant antigens is clearly required to improve the accuracy of such diagnostic tools. Our data revealed that the combination of six diagnostic proteins yielded a diagnostic sensitivity of 66.3% and specificity of 89.6%, which were significantly higher than that of the individual proteins. In addition, it can be speculated that our approach has the advantage of covering a greater extent of the *M. tuberculosis* proteome using correctly refolded proteins. This is important due to the heterogeneity of antibody responses to TB among individuals and different ethnic groups^42^.

**Figure 5:**
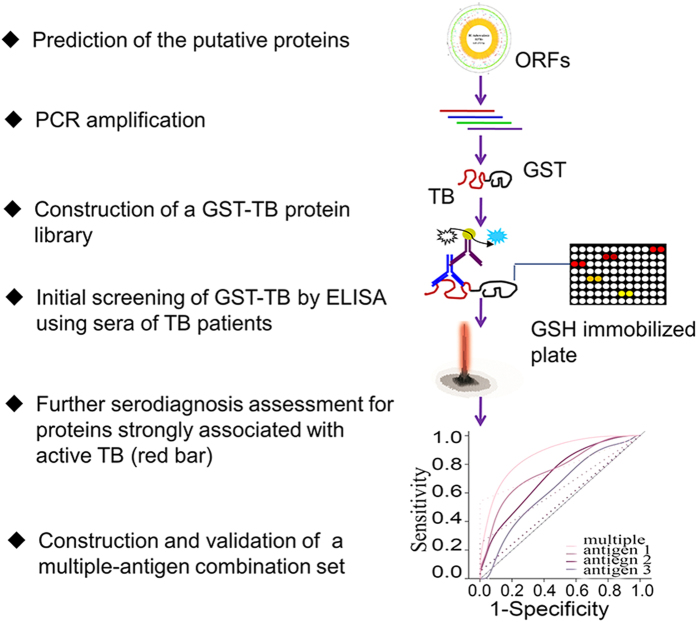
Overview of the experimental design. The putative proteins were predicted from the whole genome of *M. tuberculosis*. The selected genes were amplified by PCR and cloned into a GST-fusion protein expression vector. The fusion genes (GST-TBs) were expressed in *E. coli*. The refolded proteins were arrayed on GSH-immobilized plates and the GST-TB protein library was constructed. Serum samples from TB patients were used to probe the GST-TB arrayed microplates. Seropositive antigens were identified by ELISA. Proteins strongly associated with TB were further assessed with a panel of TB patient serum samples. A multiple-antigen combination set was predicted by R statistical software and validated.

Over the recent decades, TB immunodiagnostics including cell-immune-based diagnostic tests and serological tests were developed for diagnosing infected individuals with *M. tuberculosis*. However, majority of them had poor performances in terms of their sensitivities and specificities^17^,^45^,^46^. The cell-immune-based diagnostic tests have several irreversible drawbacks. For example, the century old TST is limited to use by its low specificity because of cross-reactivity with previous BCG vaccination and other environmental mycobacterium infection while the recently developed interferon-γ release assays (IGRAs), despite of high specificity compared with TST, is expensive and need particular expertise which presents an additional barrier in resource-limited settings. Moreover, both of the tests have the poor positive predictive value (PPV) for progression of latent tuberculosis infection (LTBI) to active TB^46^. In addition, none of two immune-based tests performs optimal sensitivity in individuals who are immunocompromised and is able to discriminate active or past disease from latent infection in areas with a high burden of TB. Serological tests represent an attractive option for diagnosing active TB. However, antibody-based diagnosis is controversial and is widely regarded as useless. WHO currently recommends against using these TB serological tests due to the suboptimal sensitivity and specificity^47^. Therefore, further identification and screening of novel serodiagnostic antigens is still highly recommended by WHO. In this study, after a stepwise selection process (represented schematically in Fig. 5), a multiple-antigen combination set was identified that consisted the six most immunoreactive diagnostic proteins (i.e. the 38 kDa antigen, LprG, Rv1566c, Rv1623c, MPT64 and HspX) for active TB. This combination set was selected through analysis of over 16,000 different combinations, which revealed higher accurate diagnosis compared to any of the other combinations and the individual antigens. Of the combined antigens, four proteins, the 38 kDa antigen, LprG, MPT64 and the LTBI-associated antigen HspX, have been reported previously as candidate diagnostic biomarkers^27^,^28^,^48^. In contrast, Rv1566c and Rv1623c were identified as novel antigens with one or more transmembrane helixes as predicted by TMHHM. A comparative study showed that the multiple-antigen combination set conferred a significant increase in diagnostic accuracy compared to a commercially available kit. Although the multiple-antigen combination set could be potentially developed as a novel serodiagnostic tool for active TB, the sensitivity and specificity of the multiple-antigen combination set still need to be improved. The diagnostic value of the combination set would be expected to be even lower in clinical practice, where TB contacts and healthy, latently-infected individuals would cloud the diagnosis. Potentially, this approach could be developed for the detection of latent and preclinical TB, which represents a reservoir for transmission. The increased sensitivity of such a diagnostic tool will provide support for the TB control program in achieving a reduction in the transmission of this disease.

In conclusion, we used an indirect ELISA-based GST-fusion protein array technique, in which each protein was represented in solution and correctly refolded, to identify fourteen highly reactive proteins with the TB patient serum samples including eight novel proteins as diagnostic candidates. Further, the six diagnostic proteins were identified from them through analysis of various combinations and constructed as a multiple-antigen combination set for diagnosis of active TB. A comparative study demonstrated that the set of novel protein markers had higher sensitivity and equivalent specificity compared with the current commercially available kit for diagnosis of active TB. The increased sensitivity of the diagnostic tool based on the combination set will provide support for the TB control program in achieving a reduction in the transmission of this disease.

## Methods

### Cloning and expression of GST-TB fusion proteins

All methods were carried out in accordance with the approved guidelines. The putative secreted and transmembrane proteins were predicted by bioinformatics tools, i.e. SignalP (http://www.cbs.dtu.dk/services/SignalP/)^49^ and TMHMM software (http://www.cbs.dtu.dk/services/TMHMM/)^50^, respectively. The RD proteins and the latent-associated proteins were selected from the relevant published data^51^,^52^,^53^. All the selected genes were amplified by PCR and expressed in *E. coli* as GST fusion proteins (the details were included in supplementary materials).

### Serum sample collection

Serum samples from TB patients were collected from Shanghai Pulmonary Hospital during the period from October 2011 to October 2014. Patients were included in the study if they fulfilled all the following criteria: all patients were diagnosed as newly treated active TB and defined according to the fourth edition of Guidelines for treatment of tuberculosis^54^. Specifically, the active TB case definition was a patient with a positive sputum culture for the *M. tuberculosis* complex or a patient who has been diagnosed as having active TB by a clinician and has been decided to treat with a full course of TB treatment according to clinical diagnostic criteria. Diagnostic criteria included bacteriological and pathological results, typical radiological manifestation and clinical response to anti-TB treatment consistent with active TB. All negative control serum samples were collected from healthy individuals with no history of TB and who tested negative by T-SPOT.TB or QFT-G. Individuals with HIV, hepatitis infections or autoimmune disorders were excluded. The serum samples used for every round of screening were independent and not recycled. Patients were tested by TB-DOT (Diagnostic Kit for Antibody to *Mycobacterium tuberculosis* (Colloidal Gold), Shanghai) and more details regarding age, gender and sputum smear were listed in Supplementary Table S4. The serum samples were pre-adsorbed with *E. coli* lysate and the GST protein to block antibodies against *E. coli* antigens and GST proteins. A mixture of 0.1 ml human serum, 3 ml bacterial lysate, 0.4 ml GST bound Glutathione Sepharose 4B beads and 1.5 ml PBS (pH 7.4) was rocked for 5 h at room temperature. After centrifugation, the adsorbed serum was stored at −20 °C for later use.

### GST-TB protein screening using TB patient sera

The ratio of RLUs of a human serum sample to GST-TBs and the GST control was calculated using the formula: R = (RLUs of GST-TB − RLUs of PBS)/(RLUs of GST − RLUs of PBS). GST-TBs with R ≥ 2 were considered to indicate seropositive reactions.

### Indirect ELISA

The 14 identified antigens were expressed in *E. coli* and purified by Ni-NTA affinity chromatography. The optimal conditions for ELISA (antigen concentration, dilution of human serum sample dilution and HRP-conjugated anti-human IgG secondary antibody dilution) were determined according to the phalanx titration principle.

### Selection of antigens for a multiple-antigen system

To overcome the poor diagnostic sensitivity of a single antigen, variable combinations of proteins (range, 2–14) were calculated using the caret package in R statistical software (http://www.r-project.org/).

## Other Materials and Methods

A detailed description of methodologies (*Cloning and expression of GST-TB fusion proteins, GST-TB arrayed plates, GST-TB protein screening using TB patient sera, Indirect ELISA*, etc.) used in this study can be found in the Supplementary materials.

### Statistical analysis

Heat maps and analysis of multiple-antigen combinations were performed with R statistical software. Sensitivity and specificity were calculated according to the following formulas: sensitivity = number of true positives/(number of true positive + number of false negatives) and specificity = number of true negatives/(number of false positives + number of true negatives).

### Ethics statement

All study procedures were conducted with Internal Review Board approval (Tongji University School of Medicine, China). All participants received information on the aim and procedures of the study, and written informed consent was obtained from all subjects.

## Additional Information

**How to cite this article**: Zhou, F. *et al.* Protein array identification of protein markers for serodiagnosis of *Mycobacterium tuberculosis* infection. *Sci. Rep.* **5**, 15349; doi: 10.1038/srep15349 (2015).

## Supplementary Material

Supplementary Information

## Figures and Tables

**Table 1:** Sensitivities and specificities of six individual antigens and the multiple-antigen combination set with sera from TB patients and healthy controls.

Rv.	Patients(N = 96)			Sensitivity(%, 95% CI)	Healthy controls(N = 49)		Specificity(%, 95% CI)
	SpecificPositive	NonspecificPositive	Negative		Positive	Negative	
Rv0934	7	23	66	31.3(22.2–41.5)	1	48	98.0(89.2–99.6)
Rv1411c	2	13	71	15.6(9.0–24.5)	2	47	95.9(86.0–99.5)
Rv1566c	3	15	78	18.8(10.7–26.8)	1	48	98.0(89.2–99.6)
Rv1623c	11	16	69	28.1(19.4–38.2)	0	49	100.0(92.8–100)
Rv1980c	7	13	76	20.8(13.2–30.3)	1	48	98.0(89.2–99.6)
Rv2031c	6	20	70	27.1(18.5–37.1)	0	49	100.0(92.8–100)
Multiple	65		31	67.7(57.4–76.9)	4	45	91.8(80.4–97.7)

Abbreviation: CI, confidence intervals.

**Table 2:** Comparison of diagnostic validity of the multiple-antigen test with the TAID-Kit.

	Truepositive	Falsenegative	Truenegative	Falsepositive	Sensitivity(%, 95% CI)*	Specificity(%, 95% CI)**	Accuracy(%)
Multiple	191	97	86	10	66.3(60.5–71.8)	89.6(83.3–95.4)	72.1
TAID-Kit	91	197	87	9	31.6(26.3–37.3)	90.6(83.0–95.6)	46.4

*p < 0.0001; **p = 1.0000 when compared by McNemar’s test. Accuracy was defined as true-positive and true-negative test results divided by the total number of specimens examined.
