# Supplementary material for: Protein array identification of protein markers for serodiagnosis of Mycobacterium tuberculosis infection
Source: Sci Rep. 2015 Oct 20;5:15349. doi: 10.1038/srep15349 (PMC4642701; doi:10.1038/srep15349)
Supplement: Supplementary Information [file srep15349-s1.pdf]

Title: Protein array identification of protein markers for serodiagnosis of *Mycobacterium tuberculosis* infection

Fangbin Zhou, Xindong Xu, Sijia Wu, Xiaobing Cui, Lin Fan*, and Weiqing Pan*

Other Materials and Methods

Supplementary Methods

Cloning and expression of GST-TB fusion proteins

The genes of selected proteins were amplified by PCR using the H37Rv strain genomic DNA as a template. The purified PCR products were cloned into the pGEX-His expression vector and recombinant proteins were expressed as a fusion protein with an N-terminal GST-tag and a C-terminal His-tag. GST-TB fusion proteins expression in *E. coli Rosetta* (DE3; Novagen, Germany) was induced with 1 mM isopropyl-β-D-thiogalactoside (IPTG) and analyzed by sodium dodecyl sulfate polyacrylamide gel electrophoresis (SDS-PAGE) and Western blotting. The inclusion bodies were denatured by the addition of 0.05 mM TCEP and 4.4% N-lauroylsarcosine and refolded in a universal refolding buffer C7 (1.0 mM TCEP, 250 mM NaCl, 12.5 mM β-cyclodextrin, 0.5 M L-arginine, 50 mM Tris-HCl pH 7.5) from the iFOLD Protein Refolding System 1 as described previously[1].

GST-TB arrayed plates

GST-TB fusion protein concentration was measured by sandwich ELISA with mouse monoclonal anti-GST antibodies and biotinylated rabbit polyclonal anti-GST-tag antibodies as previously reported[1]. Each fusion protein was adjusted to 2 μg/ml with phosphate-buffered saline (PBS) and 100 μl was added to each well of GSH-immobilized plates for incubation overnight at 4°C. Plates were washed five times with 375 μl PBST

(137 mM NaCl, 2.7 mM KCl, 10 mM Na_2HPO_4, 2 mM KH_2PO_4, and 0.05% Tween-20), and then blocked in 0.2 ml 5% skimmed milk for 2 h at room temperature. After five washes, the GST-TB arrayed plates were stored at -20 ℃. GST-TB protein binding was confirmed by ELISA with both anti-GST-tag and anti-polyhistidine-tag mouse monoclonal antibodies.

GST-TB protein screening using TB patient sera

The pre-adsorbed serum (100 µl of 1:1,000 dilution in PBST) was added to each well and incubated at 37 ℃ for 1 h. After washing five times, 100 µl of 1:20,000 diluted HRP-conjugated anti-human IgG secondary antibody (Promega, USA) was added for incubation at 37 ℃ for a further 1 h. Each well was washed five times before 100 µl of SuperSignal ELISA Femto Maximum Sensitivity Substrate solution (Pierce, USA) was added. Between 1–5 minutes after adding the substrate, the bound antibodies were quantified by measuring RLUs at 425 nm with a luminometer (SpectraMax M5, USA).

Indirect ELISA

Purified recombinant antigens were diluted (final concentration 0.5–2 µg/ml) in the coating buffer (0.05 M Na_2CO_3-$NaHCO_3$, pH 9.6) and coated on 96-well Immunosorp plates (Nunc, Denmark) overnight at 4 ℃. After blocking, test serum samples (diluted 1:50 in the blocking buffer) were added and incubated at 37 ℃ for 1 h. HRP-conjugated secondary antibody (1:10,000) was then added for 0.5 h at 37 ℃. The color reaction was

developed by TMB (3, 3′, 5, 5′-tetramethylbenzidine) substrate solution and stopped by the addition of 2 N H_2SO_4. The optical density (OD) at 450 nm was measured using a microplate reader (ELX50, Bio-Tek Instruments, USA). A positive antibody test was defined as an OD value greater than the cutoff value, i.e., the mean OD value plus three SD from the negative healthy control serum.

Supplmentary results

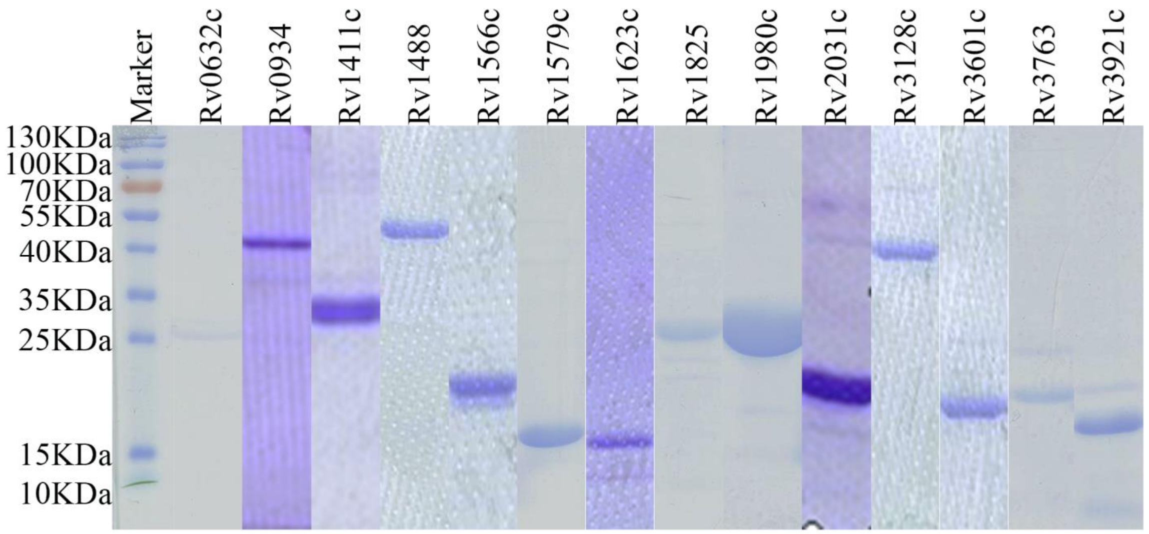

Supplementary Figure S1: Purification of 14 candidate antigens. Purified recombinant proteins were examined by SDS-PAGE and quantified using the Beyotime BCA protein quantitation kits. The purities of each protein were determined by Quality One software. The yields of the proteins were variable ranging from 0.5 mg/100 ml to 6.0 mg/100 ml 2×YT liquid medium. Of those proteins, when expressed in *E. coli*, some were mainly expressed in the supernatant, while the others were predominately expressed in inclusion bodies. For the membrane proteins, we chose only the largest extracellular domain for expression, so the actual molecular weights of those proteins, including Rv1488, Rv1566c, Rv1623c, Rv1825 and Rv1980c, were less than their theoretical values. The gels were cropped and run under the same experimental conditions.

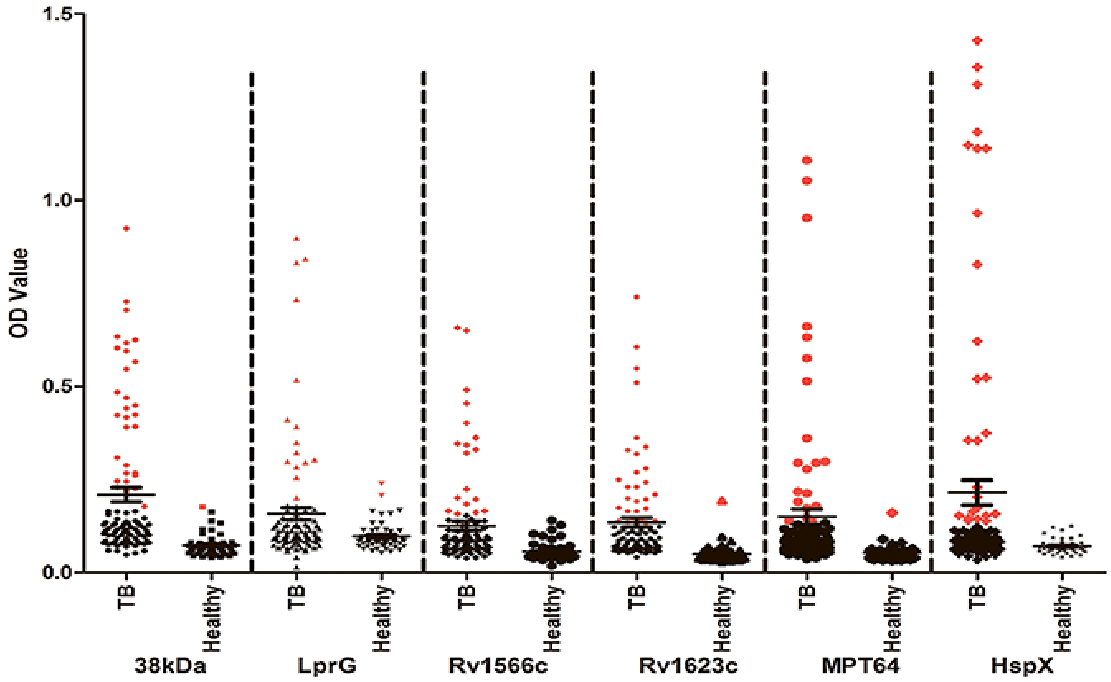

Supplementary Figure S2: Diagnostic assessment of the multiple-antigen combination set. The multiple-antigen combination set was assessed by ELISA in subjects (n = 145, 96 TB patients and 49 healthy controls). Levels of antibodies specific for the members of the multiple-antigen combination set were shown. Red dots indicate seropositive reactions. OD=optical density.

Supplementary Table S1 Classification and production of recombinant GST-TB fusion proteins from Mycobacterium *tuberculosis*.

Classification of Proteins	Predicted No.	Selected No.	Cloned No.	Seropositive
Secreted Protein	239	239	154	39 (25.3%)
Transmembrane Protein	358	240	150	31 (20.7%)
RD Protein	129	129	46	4 (4.2%)
Latent-associated Protein	91	91	91	18 (19.8%)
Total	817	699	441	92 (20.9%)

Supplementary Table S2 The behavior of the 14 proteins when expressed in *E. coli*.

Rv.	MW (actual kDa)#	MW (theoretical kDa)	Purity (%)	Yield (mg/100ml)	Folding state
Rv0632c	24.5	24.5	91.8	0.5	inclusion body
Rv0934	38.0	38.0	84.9	2.0	inclusion body
Rv1411c	25.5	25.5	92.0	3.0	inclusion body
Rv1488	39.7	41.4	95.8	4.0	inclusion body
Rv1566c	16.4	24.1	90.5	3.5	Supernatant
Rv1579c	11.3	11.3	98.1	3.0	Supernatant
Rv1623c	10.8	53.9	86.6	1.0	inclusion body
Rv1825	21.1	30.7	84.2	1.5	Supernatant
Rv1980c	22.5	24.9	86.9	6.0	inclusion body
Rv2031c	16.3	16.3	88.3	5.0	Supernatant
Rv3128c	37.7	37.7	94.8	3.0	Supernatant
Rv3601c	15.0	15.0	92.1	3.0	inclusion body
Rv3763	15.2	15.2	80.5	1.5	inclusion body
Rv3921c	11.9	11.9	85.6	3.0	Supernatant

#Abbreviation: MW, molecular weight

Supplementary Table S3 Proteins associated with active tuberculosis.

Rv.	Gene	Sensitivity (%, 95% CI)	Specificity (%, 95% CI)	Annotation
Rv0632c	EchA3	16.7 (7.0–31.4)	90.9 (70.8–98.9)	Enoyl-CoA hydratase EchA3
Rv0934	pstS1	33.3 (19.6–49.6)	95.5 (77.2–99.9)	Phosphate-binding lipoprotein PstS1
Rv1411c	LprG	14.3 (5.4–28.5)	95.5 (77.2–99.9)	Conserved lipoprotein LprG
Rv1488	-	26.2 (13.9–42.0)	95.5 (77.2–99.9)	Possible exported conserved protein
Rv1566c	-	16.7 (7.0–31.4)	100.0 (77.2–99.9)	Possible Inv protein
Rv1579c	-	11.9 (4.0–25.6)	90.9 (70.8–98.9)	Phage protein
Rv1623c	cydA	26.2 (13.9–42.0)	95.5 (84.6–100)	Cytochrome D ubiquinol oxidase CydA
Rv1825	-	11.9 (4.0–25.6)	95.5 (77.2–99.9)	Hypothetical protein
Rv1980c	MPT64	21.4 (10.3–36.8)	95.5 (77.2–99.9)	Immunogenic protein Mpt64
Rv2031c	HspX	33.3 (19.6–49.6)	100.0 (84.6–100)	Heat shock protein HspX
Rv3128c	-	9.5 (2.7–22.6)	95.5 (77.2–99.9)	Pseudo
Rv3601c	panD	21.4 (10.3–36.8)	86.4 (65.1–97.1)	Probable aspartate 1-decarboxylase precursor PanD
Rv3763	LpqH	9.5 (2.7–22.6)	95.5 (77.2–99.9)	19 kDa lipoprotein antigen precursor LpqH
Rv3921c	YidC	9.5 (2.7–22.6)	81.8 (59.7–94.8)	Membrane protein insertase YidC
Multiple-antigen	-	71.4 (55.4–84.3)	86.4 (65.1–97.1)	

Supplementary Table S4 Clinical characteristics of the study population.

Characteristic	Active TB	Healthy control
**Round one**		
Number	10	3
Age, median (range) (yr)	44.8 (22-63)	39.3 (32-47)
Male/female	5/5	2/1
sputum smear +/-	4/6	NA.[#]
Cavitary +/-	2/8	NA.
TB-DOT	6/4	NA.
Period of enrollment (m, yr)	October, 2011	
**Round two**		
Number	42	22
Age, median (range) (yr)	39.2 (17-80)	29.5 (18-61)
Male/female	31/11	14/8
sputum smear +/-	8/34	NA.
Cavitary +/-	9/33	NA.
TB-DOT	16/26	NA.
Period of enrollment (m, yr)	November, 2011- February, 2012	
**Round three**		
Number	96	49
Age, median (range) (yr)	43.5 (15-90)	34.1 (13-52)
Male/female	70/26	29/20
sputum smear +/-	19/77	NA.
Cavitary +/-	16/80	NA.
TB-DOT	32/64	NA.
Period of enrollment (m, yr)	March, 2012- August, 2012	
**Round four**		
Number	288	96
Age, median (range) (yr)	44.2 (13-91)	42.6 (9-78)
Male/female	207/81	52/44
sputum smear +/-	67/221	NA.
Cavitary +/-	56/232	NA.
TB-DOT	121/167	NA.
Period of enrollment (m, yr)	September, 2012-October, 2014	

[#] Abbreviation: N.A., not applicable

Supplementary Table S5 The 432 expressed GST-TB fusion proteins detected by SDS-PAGE and Western blot.

No.	Rv#
1–12	Rv0014c Rv0015c Rv0016c Rv0039c Rv0040c Rv0051 Rv0052 Rv0072 Rv0079 Rv0080 Rv0081 Rv0096-1
13–24	Rv0096-2 Rv0109 Rv0116c Rv0124 Rv0129c Rv0144 Rv0152c Rv0169 Rv0170 Rv0174 Rv0175 Rv0176
25–36	Rv0178 Rv0179c Rv0195 Rv0200 Rv0202c Rv0202c-1 Rv0202c-2 Rv0203 Rv0218 Rv0221 Rv0226c Rv0227c
37–48	Rv0229c Rv0236c-1 Rv0236c-2 Rv0237 Rv0251c Rv0265c Rv0278c Rv0284 Rv0285 Rv0288 Rv0291 Rv0309
49–60	Rv0315 Rv0335c Rv0342 Rv0344c Rv0347 Rv0361 Rv0398c Rv0399c Rv0402c-1 Rv0402c-2 Rv0403c Rv0411c
61–72	Rv0418 Rv0419 Rv0425c Rv0432 Rv0436c-1 Rv0436c-2 Rv0455c Rv0476 Rv0479c Rv0490 Rv0505c Rv0506
73–84	Rv0550c Rv0528 Rv0538 Rv0559c Rv0565c Rv0569 Rv0569 Rv0571C Rv0572c Rv0578c Rv0583c Rv0585c
85–96	Rv0592 Rv0603 Rv0604 Rv0632c Rv0640 Rv0671 Rv0674 Rv0676c Rv0677c Rv0680c Rv0700 Rv0710
97–108	Rv0713 Rv0732 Rv0736 Rv0747 Rv0779c Rv0792c Rv0817c Rv0826 Rv0835 Rv0838 Rv0865 Rv0867c
109–120	Rv0875c Rv0899 Rv0902c Rv0928 Rv0932c Rv0934 Rv0962c Rv978c Rv980c Rv0982 Rv0987-1 Rv0987-2
121–132	Rv0990c Rv1004c Rv1006 Rv1032c Rv1075c Rv1078 Rv1081c Rv1096 Rv1097c Rv1100 Rv1115Rv1132
133–144	Rv1145 Rv1146 Rv1152 Rv1158c Rv1166 Rv1214c Rv1221 Rv1223 Rv1233c Rv1252c Rv1269c Rv1270c
145–156	Rv1275 Rv1284 Rv1290c Rv1296 Rv1348-1 Rv1348-2 Rv1352 Rv1362c Rv1411c Rv1418 Rv1419Rv1430
157–168	Rv1431 Rv1433 Rv1435c Rv1471 Rv1477 Rv1488 Rv1506c Rv1507c Rv1510 Rv1513 Rv1514c Rv1515c
169–180	Rv1521 Rv1528c Rv1541c Rv1557 Rv1565c Rv1566c Rv1573 Rv1574 Rv1576c Rv1577c Rv1578c Rv1579c
181–192	Rv1580c Rv1582c Rv1584c Rv1585c Rv1586c Rv1592c Rv1614 Rv1623c Rv1698 Rv1732c Rv1733c Rv1734c
193–204	Rv1738 Rv1743 Rv1746 Rv1766 Rv1767 Rv1771 Rv1773c Rv1779c Rv1782 Rv1788 Rv1791Rv1793
205–216	Rv1804c Rv1805c Rv1810 Rv1812c Rv1813c Rv1815 Rv1825 Rv1841c Rv1842c Rv1860 Rv1881c Rv1885c
217–228	Rv1886c Rv1899c Rv1906c Rv1911c Rv1921c Rv1922 Rv1926c Rv1926c Rv1967 Rv1968 Rv1969Rv1971
229–240	Rv1972 Rv1974 Rv1975 Rv1977 Rv1980c Rv1980c Rv1984c Rv1987 Rv1988 Rv1996 Rv1998c Rv2003c
241–252	Rv2005c Rv2005c Rv2007c Rv2012 Rv2016 Rv2017 Rv2028c Rv2029c Rv2030c Rv2030c Rv2031c Rv2031c
253–264	Rv2080 Rv2107 Rv2138 Rv2151c Rv2194 Rv2224c Rv2253 Rv2262c Rv2270 Rv2290 Rv2293c Rv2301
265–276	Rv2307c Rv2339 Rv2345 Rv2346c Rv2347c Rv2348c Rv2349c Rv2351c Rv2366c Rv2376c Rv2403c Rv2415c

No.	Rv#
277–288	Rv2428 Rv2434c Rv2443 Rv2450c Rv2469c Rv2497c Rv2515c Rv2518c Rv2553c Rv2563 Rv2575 Rv2582
289–300	Rv2586c Rv2587c Rv2601 Rv2623 Rv2623 Rv2625c Rv2626c Rv2628 Rv2629 Rv2630 Rv2631Rv2645
301–312	Rv2646 Rv2647 Rv2648 Rv2651c Rv2652c Rv2654c Rv2656c Rv2657c Rv2658c Rv2659c Rv2660c Rv2661c
313–324	Rv2668 Rv2672 Rv2673 Rv2690c Rv2699c Rv2706c Rv2719c Rv2728c Rv2745c Rv2784c Rv2864c Rv2873
325–336	Rv2875 Rv2891 Rv2905 Rv2911 Rv2972c Rv2999 Rv3000 Rv3004 Rv3016 Rv3018c Rv3021c Rv3033
337–348	Rv3034c Rv3036c Rv3044 Rv3054c Rv3081 Rv3090 Rv3092c Rv3103c Rv3106 Rv3117 Rv3118 Rv3121
349–360	Rv3126c Rv3128c Rv3129 Rv3130c Rv3130c Rv3132c Rv3133c Rv3134c Rv3194c Rv3206c Rv3239c Rv3267
361–372	Rv3271c Rv3276c Rv3291c Rv3291c Rv3310 Rv3330 Rv3334 Rv3340 Rv3354 Rv3390 Rv3400Rv3404c
373–384	Rv3405c Rv3413c Rv3428c Rv3435c Rv3450c Rv3452 Rv3468c Rv3482c Rv3491 Rv3492c Rv3493c Rv3497c
385–396	Rv3524 Rv3572 Rv3574 Rv3576 Rv3584 Rv3593 Rv3601c Rv3619c Rv3622c Rv3627c Rv3671c Rv3683
397–408	Rv3693 Rv3695 Rv3705c Rv3706c Rv3717 Rv3728 Rv3732 Rv3737 Rv3738c Rv3759c Rv3763Rv3764c
409–420	Rv3779 Rv3802c Rv3803c Rv3804c Rv3804c Rv3805c Rv3807c Rv3826-1 Rv3826-2 Rv3833 Rv3835Rv3841
421–432	Rv3842c Rv3848 Rv3849 Rv3852 Rv3870 Rv3875 Rv3875 Rv3882c Rv3883c Rv3885c Rv3908 Rv3921c

Supplementary Table S6 Seropositive *Mycobacterium tuberculosis* proteins.

Rv #	Gene	Annotation	Reported	Reference
Rv0072	-	Probable glutamine-transport transmembrane protein ABC transporter	-	-
Rv0096	PPE1	PPE family protein PPE1	-	-
Rv0116c	ldtA	Probable L,D-transpeptidase LdtA	-	-
Rv0129c	fbpC	Secreted antigen 85-C FbpC (85C) (antigen 85 complex C) (AG58C)	+	2
Rv0144	-	Probable transcriptional regulatory protein (possibly TetR-family)	-	-
Rv0170	mce1B	Mce-family protein Mce1B	-	-
Rv0174	mce1F	Mce-family protein Mce1F	-	-
Rv0203	-	Possible exported protein	-	-
Rv0237	-	Probable conserved lipoprotein LpqI	-	-
Rv0251c	hsp	Heat shock protein Hsp	+	3,4
Rv0265c	-	Probable periplasmic iron-transport lipoprotein	-	-
Rv0284	eccC3	ESX conserved component EccC3 ESX-3 type VII secretion system protein Possible membrane protein	-	-
Rv0285	PPE	PE family protein PE5	-	-
Rv0309	-	Possible conserved exported protein	-	-
Rv0335c	PE6	PE family protein PE6	-	-
Rv0403c	MmpS1	Probable conserved membrane protein MmpS1	-	-
Rv0432	sodC	Periplasmic superoxide dismutase [Cu-Zn] SodC	-	-
Rv0436c	pssA	Probable CDP-diacylglycerol--serine O-phosphatidyltransferasePssA	-	-
Rv0506	mmpS2	Probable conserved membrane protein MmpS2	-	-
Rv0569	-	hypothetical protein	-	-
Rv0632c	panD	Probable aspartate 1-decarboxylase precursor PanD	+	2
Rv0674	-	hypothetical protein	-	-
Rv0677c	mmpS5	Possible conserved membrane protein MmpS5		-
Rv0700	rpsJ	30S ribosomal protein S10 RpsJ	-	-

Rv #	Gene	Annotation	Reported	Reference
Rv0835	lpqQ	Possible lipoprotein LpqQ	-	-
Rv0865	mog	Probable molybdopterin biosynthesis Mog protein	-	-
Rv0934	pstS1	Periplasmic phosphate-binding lipoprotein PstS1 (PBP-1) (PstS1)	+	5-7
Rv0990c	Hsp22.5	Hypothetical protein	-	-
Rv1075c	-	Conserved exported protein	-	-
Rv1115	-	Possible exported protein	-	-
Rv1146	mmpL13b	Probable conserved transmembrane transport protein MmpL13b	-	-
Rv1166	LpqW	Probable conserved lipoprotein LpqW	-	-
Rv1221	sigE	Alternative RNA polymerase sigma factor SigE	-	-
Rv1223	htrA	Probable serine protease HtrA (DEGP protein)	-	-
Rv1233c	-	hypothetical protein	-	-
Rv1275	lprC	Possible lipoprotein LprC	-	-
Rv1296	thrB	Probable homoserine kinase ThrB	-	-
Rv1411c	LprG	Conserved lipoprotein LprG	+	3
Rv1418	LprH	Probable lipoprotein LprH	-	-
Rv1433	Rv1433	Possible conserved exported protein	-	-
Rv1471	trxB1	Probable thioredoxin TrxB1	-	-
Rv1488	-	Possible exported conserved protein	-	-
Rv1521	fadD25	Probable fatty-acid-AMP ligase FadD25	-	-
Rv1566c	-	Possible Inv protein	-	-
Rv1574	-	Probable PhiRv1 phage related protein	-	-
Rv1578c	-	Probable PhiRv1 phage protein	-	-
Rv1579c	-	Probable PhiRv1 phage protein	-	-
Rv1592c	-	hypothetical protein	-	-
Rv1614	lgt	Possible prolipoproteindiacylglyceryltransferasesLgt	-	-
Rv1623c	cydA	Probable integral membrane cytochrome D ubiquinol oxidase CydA	-	-

Rv #	Gene	Annotation	Reported	Reference
Rv1804c	-	Hypothetical protein	+	8
Rv1810	-	Hypothetical protein	-	-
Rv1815	-	Hypothetical protein	-	-
Rv1825	-	Hypothetical protein	-	-
Rv1885c	-	Chorismatemutase	-	-
Rv1906c	-	Hypothetical protein	-	-
Rv1926c	mpt63	Immunogenic protein Mpt63	-	9
Rv1967	mce3B	Mce-family protein Mce3B		-
Rv1968	mce3C	Mce-family protein Mce3C		-
Rv1977	-	Hypothetical protein	-	-
Rv1980c	mpt64	Immunogenic protein Mpt64	+	10
Rv1987	-	Possible chitinase	+	11
Rv1988	erm(37)	Probable 23S rRNAmethyltransferaseErm(37)	-	-
Rv1998c	-	hypothetical protein	-	-
Rv2005c	-	Universal stress protein family protein	-	-
Rv2007c	-	FerredoxinFdxA	+	-
Rv2012	-	hypothetical protein	-	-
Rv2016	-	Hypothetical protein	-	-
Rv2031c	hspX	Heat shock protein HspX (14 kDa antigen) (HSP16.3)	+	3,12
Rv2194	qcrC	Probable ubiquinol-cytochrome C reductase QcrC (cytochrome C subunit)	-	-
Rv2307c	-	hypothetical protein	-	-
Rv2330c	IppP	Probable lipoprotein LppP	-	-
Rv2515c	-	Hypothetical protein	-	-
Rv2575	-	Possible conserved membrane glycine rich protein	-	-
Rv2582	ppiB	Probable peptidyl-prolylcis-trans isomerase B PpiB	-	-
Rv2587c	secD	Probable protein-export membrane protein SecD	-	-

Rv #	Gene	Annotation	Reported	Reference
Rv2623	TB31.7	Universal stress protein family protein TB31.7	+	13
Rv2626c	-	Hypoxic response protein 1 Hrp1	-	-
Rv2629	-	Hypothetical protein	-	-
Rv2630	-	Hypothetical protein	+	14
Rv2661c	-	Hypothetical protein	-	-
Rv2741	PE_PGRS47	PE-PGRS family protein PE_PGRS47	-	-
Rv2745c	clgR	Transcriptional regulatory protein ClgR	-	-
Rv2875	mpt70	Major secreted immunogenic protein Mpt70	+	15
Rv3021c	PPE47	Pseudo	-	-
Rv3033	-	Hypothetical protein	-	-
Rv3036c	TB22.2	Probable conserved secreted protein TB22.2	-	-
Rv3103c	-	Hypothetical proline-rich protein	-	-
Rv3106	fprA	NADPH:adrenodoxinoxidoreductase FprA	-	-
Rv3128c	pseudo	Pseudo	-	-
Rv3130c	tgs1	Triacylglycerol synthase (diacylglycerolacyltransferase) Tgs1	-	-
Rv3133c	devR	Two component transcriptional regulatory protein DevR	+	-
Rv3206c	moeB1	Probable molybdenum cofactor biosynthesis protein MoeB1	-	-
Rv3271c	-	Probable conserved integral membrane protein	-	-
Rv3291c	lrpA	Probable transcriptional regulatory protein LrpA (Lrp/AsnC-family)	-	-
Rv3330	DacB1	Probable penicillin-binding protein DacB1	-	-
Rv3340	-	Probable O-acetylhomoserinesulfhydrylaseMetC	-	-
Rv3354	-	Hypothetical protein	-	-
Rv3390	lpqD	Probable conserved lipoprotein LpqD	-	-
Rv3428c	-	Possible transposase	-	-
Rv3435c	-	Probable conserved transmembrane protein	-	-
Rv3450c	eccB4	ESX conserved component EccB4 ESX-4 type VII secretion system protein	-	-

Rv #	Gene	Annotation	Reported	Reference
		probable membrane protein		
Rv3452	cut4	Probable cutinase precursor Cut4	+	16
Rv3576	lppH	Possible conserved lipoprotein LppH	-	-
Rv3601c	panD	Probable aspartate 1-decarboxylase precursor PanD (aspartate alpha-decarboxylase)	-	-
Rv3693	-	Possible conserved membrane protein	-	-
Rv3695	-	Possible conserved membrane protein	-	-
Rv3738c	PPE66	PPE family protein PPE66	-	-
Rv3763	LpqH	19 kDa lipoprotein antigen precursor LpqH	+	3
Rv3803c	fbpD	Secreted MPT51/MPB51 antigen protein FbpD	+	17
Rv3804c	fbpA	Secreted antigen 85-a FbpA (mycolyltransferase 85A)	+	18
Rv3805c	aftB	Possible arabinofuranosyltransferaseAftB	-	-
Rv3807c	-	Possible conserved transmembrane protein	+	11
Rv3841	bfrB	BacterioferritinBfrB	-	-
Rv3849	EspR	ESX-1 transcriptional regulatory protein EspR	-	-
Rv3852	-	Possible histone-like protein Hns	-	-
Rv3875	esxA	6 kDa early secretory antigenic target EsxA (ESAT-6)	+	3
Rv3882c	eccE1	ESX conserved component EccE1 ESX-1 type VII secretion system protein Possible membrane protein	-	-
Rv3908	mutT4	Possible mutator protein MutT4	-	-
Rv3921c	-	Probable conserved transmembrane protein	-	-

REFERENCE

1. Xu, X. *et al.* Serodiagnosis of Schistosoma japonicum infection: genome-wide identification of a protein marker, and assessment of its diagnostic validity in a field study in China. *Lancet Infect Dis.* **14**, 489-97 (2014).
2. Kumar, G. *et al.* Diagnostic potential of Ag85C in comparison to various secretory antigens for childhood tuberculosis. *Scandinavian Journal of Immunology.* **68**, 177-183 (2008).
3. Kunnath-Velayudhan, S. *et al.* Dynamic antibody responses to the Mycobacterium tuberculosis proteome. *Proc Natl Acad Sci U S A.* **107**, 14703-8 (2010).
4. Kaushik, A. *et al.* Diagnostic potential of 16 kDa (HspX, alpha-crystalline) antigen for serodiagnosis of tuberculosis. *Indian J Med Res.* **135**, 771-7 (2012).
5. Chiappini, E. *et al.* Potential role of M. tuberculosis specific IFN-gamma and IL-2 ELISPOT assays in discriminating children with active or latent tuberculosis. *PLoS One.* **7**, e46041 (2012).
6. Rao, P.V.R. *et al.* Improved diagnosis of tuberculosis in HIV-positive patients using RD1-encoded antigen CFP-10. *International Journal of Infectious Diseases.* **13**, 613-622 (2009).
7. Shin, A.R. *et al.* Improved sensitivity of diagnosis of tuberculosis in patients in Korea via a cocktail enzyme-linked immunosorbent assay containing the abundantly expressed antigens of the K strain of Mycobacterium tuberculosis. *Clin Vaccine Immunol.* **15**, 1788-95 (2008).
8. Ben Amor, Y. *et al.* Immunological characterization of novel secreted antigens of Mycobacterium tuberculosis. *Scand J Immunol.* **61**, 139-46 (2005).
9. Mustafa, A.S. Th1 cell reactivity and HLA-DR binding prediction for promiscuous recognition of MPT63 (Rv1926c), a major secreted protein of Mycobacterium tuberculosis. *Scand J Immunol.* **69**, 213-22 (2009).
10. Araujo, L.S., Maciel, R.M., Trajman, A. & Saad, M.H. Assessment of the IgA immunoassay diagnostic potential of the Mycobacterium tuberculosis MT10.3-MPT64 fusion protein in tuberculous pleural fluid. *Clin Vaccine Immunol.* **17**, 1963-9 (2010).
11. Li, Y. *et al.* A proteome-scale identification of novel antigenic proteins in Mycobacterium tuberculosis toward diagnostic and vaccine development. *J Proteome Res.* **9**, 4812-22 (2010).
12. Zhang, L. *et al.* Identification of putative biomarkers for the serodiagnosis of drug-resistant Mycobacterium tuberculosis. *Proteome Sci.* **10**, 12 (2012).
13. Jain, R.K. *et al.* Mycobacterial Dormancy Regulon Protein Rv2623 as a Novel Biomarker for the Diagnosis of Latent and Active Tuberculous Meningitis. *Disease Markers*. 311-316 (2013).
14. Kassa, D. *et al.* Analysis of Immune Responses against a Wide Range of Mycobacterium tuberculosis Antigens in Patients with Active Pulmonary Tuberculosis. *Clinical and Vaccine Immunology.* **19**, 1907-1915 (2012).
15. Windish, H.P. *et al.* Protection of mice from Mycobacterium tuberculosis by ID87/GLA-SE, a novel tuberculosis subunit vaccine candidate (vol 29, pg 7842, 2011). *Vaccine.* **31**, 278-278 (2012).
16. Brust, B. *et al.* Mycobacterium tuberculosis Lipolytic Enzymes as Potential Biomarkers for the Diagnosis of Active Tuberculosis. *Plos One.* **6**(2011).
17. Bethunaickan, R., Baulard, A.R., Locht, C. & Raja, A. Antibody response in pulmonary tuberculosis against recombinant 27kDa (MPT51, Rv3803c) protein of Mycobacterium tuberculosis.

Scandinavian Journal of Infectious Diseases. **39**, 867-874 (2007).

18. Gartner, T. *et al.* Mucosal prime-boost vaccination for tuberculosis based on TLR triggering OprI lipoprotein from Pseudomonas aeruginosa fused to mycolyl-transferase Ag85A. *Immunology Letters.* **111**, 26-35 (2007).
